# Hybrid Formulations of Liposomes and Bioadhesive Polymers Improve the Hypotensive Effect of the Melatonin Analogue 5-MCA-NAT in Rabbit Eyes

**DOI:** 10.1371/journal.pone.0110344

**Published:** 2014-10-20

**Authors:** Daniela Quinteros, Marta Vicario-de-la-Torre, Vanessa Andrés-Guerrero, Santiago Palma, Daniel Allemandi, Rocío Herrero-Vanrell, Irene T. Molina-Martínez

**Affiliations:** 1 Department of Pharmacy, Facultad de Ciencias Químicas, Universidad Nacional de Córdoba, CONICET, Edificio de Ciencias II, Ciudad Universitaria, Córdoba, Argentina; 2 Department of Pharmacy and Pharmaceutical Technology, Faculty of Pharmacy, Complutense University of Madrid, Pharmaceutical Innovation in Ophthalmology Research Group, Sanitary Research Institute of the San Carlos Clinical Hospital (IdISSC) and the Ocular Pathology National Net (OFTARED) of the Institute of Health Carlos III, Madrid, Spain; Casey Eye Institute, United States of America

## Abstract

For the treatment of chronic ocular diseases such as glaucoma, continuous instillations of eye drops are needed. However, frequent administrations of hypotensive topical formulations can produce adverse ocular surface effects due to the active substance or other components of the formulation, such as preservatives or other excipients. Thus the development of unpreserved formulations that are well tolerated after frequent instillations is an important challenge to improve ophthalmic chronic topical therapies. Furthermore, several components can improve the properties of the formulation in terms of efficacy. In order to achieve the mentioned objectives, we have developed formulations of liposomes (150–200 nm) containing components similar to those in the tear film and loaded with the hypotensive melatonin analog 5-methoxycarbonylamino-N-acetyltryptamine (5-MCA-NAT, 100 µM). These formulations were combined with mucoadhesive (sodium hyaluronate or carboxymethylcellulose) or amphiphilic block thermosensitive (poloxamer) polymers to prolong the hypotensive efficacy of the drug. In rabbit eyes, the decrease of intraocular pressure with 5-MCA-NAT-loaded liposomes that were dispersed with 0.2% sodium hyaluronate, 39.1±2.2%, was remarkably higher compared to other liposomes formulated without or with other bioadhesive polymers, and the effect lasted more than 8 hours. According to the results obtained in the present work, these technological strategies could provide an improved modality for delivering therapeutic agents in patients with glaucoma.

## Introduction

Ophthalmic drug delivery is one of the most interesting and challenging endeavors facing the pharmaceutical sciences. The anatomy, physiology and biochemistry of the eye render this organ exquisitely impervious to foreign substances [1]. So to get the desired therapeutic effect, repeated and frequent applications of topical ophthalmic formulations are usually required.

Glaucoma is a multifactorial, progressive and neurodegenerative disease characterized by atrophy of the optic nerve and loss of retinal ganglion cells that can eventually lead to loss of visual acuity and visual field [2], [3]. High intraocular pressure (IOP) is considered the greatest risk factor for the development of glaucoma, so most treatments consist of the chronic application of eye drops containing hypotensive agents. Despite the usefulness of topical administration, glaucoma treatments are usually associated with adverse reactions generated by the frequent exposure to drugs and excipients undergone by the eye. Among the excipients, preservatives can induce ocular surface alterations [4], [5], [6], [7] that contribute to the development of secondary ophthalmic diseases such as the dry eye syndrome [8], [9]. This in turn can compromise patient compliance. However, the elimination of preservatives from ophthalmic formulations is not always enough to avoid side effects on the ocular surface. For that reason, the incorporation of new components in formulations with beneficial properties for the eye and able, at the same time, to increase the bioavailability of the drug, results of great interest in this field.

Melatonin receptors have been identified in the cornea, ciliary body, lens, choroid, and sclera and play a role in aqueous humor dynamics [10], [11]. In recent years, the role of melatonin and its analogues in the control of IOP has been investigated. The melatonin analogue 5-methoxycarbonylamino-N-acetyltryptamine (5-MCA-NAT) induces an IOP reduction after topical administration in normotensive rabbits [12], [13] and glaucomatous monkeys [14]. In a previous work, the hypotensive effect of 5-MCA-NAT was enhanced up to 30% (maximum IOP reduction) with the use of mucoadhesive polymers, although the effect could not be prolonged longer than 6 hours [15].

Ophthalmic formulations of liposomes have been proposed to increase the efficacy of topical ophthalmic formulations and, depending on composition, to resemble the tear film lipid layer. Liposomes are spherical vesicles composed of an aqueous core enclosed by concentric phospholipids bilayers [16]. On the ocular surface, these vesicles establish a very tight contact with tissues, thus increasing the residence time of the formulation and, subsequently, the corneal penetration of the drug [17]. When active substances are included in these systems they are protected from the medium and the half-life is usually increased.

Our research group recently developed a novel artificial tear based on liposomes that improve the quality and stability of an unstable tear film [18]. In this formulation, liposomes were dispersed in aqueous solutions of polymers with mucoadhesive properties. The addition of these polymers had positive effects attributed to their rheological and biophysical properties, which are similar to those of mucins involved in the maintenance of the tear film on the ocular surface. Bioadhesive polymers also provide long-lasting hydration and lubrication of the ocular surface while minimizing the loss of formulation through the nasolacrimal drainage pathway [19].

Other materials, such as thermosensitive amphiphilic block copolymers, namely poly (ethylene oxide)–poly (propylene oxide)–poly (ethylene oxide) (PEO–PPO–PEO, poloxamers), have been recently investigated in the design of novel drug delivery systems. When poloxamers are in solution, they form micelles and, depending on the concentration and temperature, are able to self-organize and transform into a viscous gel, allowing a controlled release of the drug [20].

In the present study we have used hybrid combinations of liposomes, mucoadhesive or thermosensitive polymers, and the intraocular hypotensive agent 5-MCA-NAT. These pharmaceutical formulations were characterized in terms of physicochemical properties, *in vitro* release of 5-MCA-NAT, *in vivo* tolerance and *in vivo* intraocular pressure reduction, after topical ophthalmic application in nonsedated normotensive rabbits.

## Materials and Methods

### Materials

The melatonin agonist 5-MCA-NAT was purchased from Tocris Bioscience (Bristol, UK) and 1.2-propylene glycol (PG) from Guinama (Valencia, Spain). Sodium hyaluronate (SH, Mw 400,000–800,000 g/mol) and sodium carboxymethylcellulose (CMC, 400–800 cps 2% solution at 20°C) were purchased from Abarán Materias Primas S.L. (Madrid, Spain). Phospholipon 90G purified from soy lecithin (>95% phosphatidylcholine, PC) was purchased from Phospholipid GmbH (Cologne, Germany). Cholesterol (Cht) and α-tocopherol (vitE) were acquired from Sigma Chemical Co. (St. Louis, MO, USA). Isotonic NaCl solution was prepared with ultrapure Milli-Q water (EMD Milllipore, Darmstadt, Germany). Poloxamer 407 (PX407) and Poloxamer 188 (PX188) were gifts from BASF S.A. (Buenos Aires, Argentina).

### Methods

#### 1. Quantitation of 5-MCA-NAT by high pressure liquid chromatography (HPLC)

Quantitative analyses of 5-MCA-NAT were performed with a HPLC instrument (Gilson, Middleton, WI, USA) composed of a solvent delivery pump (305 model), UV-visible detector (118 model), and controller software (UniPoint, all by Gilson). The injector was equipped with a 20 µl loop (7125 Rheodyne, Berkeley, CA, USA). The chromatographic separation was achieved as described previously [12] by a reversed phase protocol with a Mediterranea Sea18 column (25 cm×4 mm, 5 µm particle size; Teknokroma, Barcelona, Spain). The mobile phase was a mixture of methanol (Panreac, Barcelona, Spain) and ultrapure milliQ water (40∶60 v/v). The flow rate was set at 0.8 ml/min and the eluent was monitored at 244 nm.

#### 2. Sample preparation

Five formulations (Table 1) containing 100 µM 5-MCA-NAT were prepared: F1, aqueous solution of 5-MCA-NAT; F2, 5-MCA-NAT-loaded liposomes; F3, 5-MCA-NAT-loaded liposomes with 0.2% SH; F4, 5-MCA-NAT-loaded liposomes with 0.5% CMC; and F5, 5-MCA-NAT-loaded liposomes with PX (PX407 and PX188; 12/8, w/w).

**Table 1:** Nomenclature and composition of the 5-MCA-NAT formulations.

Formulation	Composition	Polymer
F1	100 µM 5-MCA-NAT, 0.275% PG and 0.788% NaCl	-
F2	100 µM 5-MCA-NAT, 0.275% PG, 10 mg/mL PC, and0.788% NaCl	-
F3	100 µM 5-MCA-NAT, 0.275% PG, 10 mg/mL PC, 0.2% SH and0.788% NaCl	Sodium hyaluronate
F4	100 µM 5-MCA-NAT, 0.275% PG, 10 mg/mL PC, 0.5% CMC and0.788% NaCl	Carboxymethylcellulose
F5	100 µM 5-MCA-NAT, 0.275% PG, 10 mg/mL PC, PX407 andPX188 (12/8 w/w), and 0.479% NaCl	Poloxamer

5-MCA-NAT: 5-methoxycarbonylamino-N-acetyltryptamine, PG: propylene glycol, PC: phosphatidylcholine, SH: sodium hyaluronate, CMC: carboxymethylcellulose, PX: poloxamer (PX407 and PX188 12/8 w/w).

Formulation F1, the aqueous non-liposomal solution of 5-MCA-NAT, was prepared by dissolution of 5-MCA-NAT in PG (10 mg/mL) and further dilution with an aqueous solution of NaCl. The final formulation contained 100 µM 5-MCA-NAT, 0.275% PG, and 0.788% NaCl. 5-MCA-NAT-loaded liposomes (F2, F3, F4, and F5) were prepared following the procedure described by Bangham [21] and modified by Vicario-de-la-Torre [22]. According to the procedure PC, Cht and vitE (8∶1∶0.08) were dissolved in chloroform in a round-bottom flask. Then, the organic solvent was slowly removed at 33°C with a rotator evaporator to produce a thin film of dry lipids on the inner surface of the flask. The dry film was then hydrated with an aqueous solution of 200 µM 5-MCA-NAT to produce the 5-MCA-NAT-loaded liposomes. The liposomes were extruded ten times through 0.22 µm pore polycarbonate membranes (Nucleopore Lipex Biomembrane, Vancouver, Canada). To ensure full lipid hydration, vesicles were allowed to mature overnight under refrigeration. After that, stock formulations were diluted (1∶2) with different aqueous solutions to achieve the final liposomal formulations (Table 1): F2, 0.788% NaCl and 0.275% PG; F3, 0.4% SH in 0.788% NaCl and 0.275% PG; F4, 1% CMC in 0.788% NaCl and 0.275% PG); F5, PX407 and PX188 (24/16, w/w) in 0.17% NaCl and 0.275% PG. The concentration of NaCl was adjusted to attain osmolarity values in the final formulations within an acceptable range for ophthalmic administration [23]. Due to the high influence of PX in the osmolarity of the formulations [24], a lower concentration of NaCl was used to adjust F4. Final liposomal formulations were sterilized by filtration through 0.22 µm pore membranes.

#### 3. Determination of the encapsulation efficiency of liposomes

The encapsulation efficiency of 5-MCA-NAT in the liposomes was determined upon separation by centrifugation (18,000 rpm, 4°C, 20 min) from the dispersing medium containing non-encapsulated 5-MCA-NAT. The amount of free 5-MCA-NAT in the supernatant was determined in triplicate by HPLC. The determination of the percentage of drug loading (PDL) in liposomes, expressed as mean ± standard deviation, was calculated using the following equation [25]:
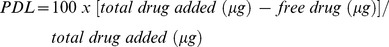



The entire amount of 5-MCA-NAT in the liposomal formulations was measured by dissolving liposomes with the appropriate amount of acetonitrile. Solutions were then centrifuged (15,000 rpm, 4°C, 20 min) and the supernatant analyzed in triplicate by HPLC.

#### 4. Determination of pH, osmolarity, and viscosity

The pH of the formulations was measured with a calibrated pH meter (model 230; Mettler, Barcelona, Spain) equipped with an InLab Microelectrode (Mettler). Measurements were performed in triplicate at room temperature (25°C).

Osmolarity was analyzed by a vapor pressure osmometer (model K-7000 Knauer, Berlin, Germany). Before performing the analyses, the osmometer was calibrated with a solution of NaCl (400 mOsm). The determinations were made in triplicate at 33°C (ocular surface temperature) [26].

Viscosity of liposomal samples was assessed in triplicate at 33°C with a thermostatically controlled rheometer (HaakeRheostress R1, Düsseldorf, Germany) using a parallel plate geometry (diameter 60 mm, gap 0.5 mm). The viscosity was calculated at a shear rate of 100 s^−1^, which was within the linear viscoelastic range for all of the formulations.

#### 5. Determination of particle size and zeta potential

Measurements of liposome particle size and zeta potential were carried out by photon correlation spectroscopy (PCS, Zetatrac, Largo, FL, USA). For the analyses, formulations were diluted 1/20 (v/v) in an aqueous medium. All determinations were performed in triplicate at room temperature (25°C).

#### 6. Hypotensive efficacy studies *in* *vivo*: IOP determinations

In this work, experiments were performed in both eyes of nonsedated normotensive male New Zealand white rabbits (2–2.5 kg). Each formulation was evaluated in 10 animals (n = 20 eyes) and each control in 5 (n = 10 eyes). In order to reduce the number of rabbits, the same animals were used multiple times as experimental and control. The animals were kept in individual cages with free access to food and water and maintained in a controlled 12/12 h light/dark cycle. All of the protocols herein were approved by the Ethics Committee for Animal Research of Complutense University of Madrid. Also, animal manipulations followed institutional guidelines, European Union regulations for the use of animals in research, and the ARVO (Association for Research in Vision and Ophthalmology) statement for the use of animals in ophthalmic and vision research.

IOP was measured with a Tonovet rebound tonometer (Tiolat, Helsinki, Finland). With this technique IOP is assessed without the need of topical anesthesia. For each eye, IOP was set at 100% with two basal readings taken 30 min before and immediately before the instillation. Then a single dose of the formulation (25 µL) was applied to both eyes. IOP determinations were performed once every hour over the next 8 hours. As a control, rabbits received formulations without the hypotensive agent. The administration protocol included a washout period of at least 48 hours between experiments.

#### 7. *In vitro* drug release

The release of 5-MCA-NAT from the different formulations was studied using a dialysis method. To do this, a dialisys membrane (Spectra/Por Float-A-Lyzer G2; 20,000 MW cut off; Iberlabo, Madrid, Spain) was employed (0.8 mL of sample). Three membranes were prepared for each formulation. A conventional solution of 100 µM 5-MCA-NAT without liposomes was used as reference. Dialysis membranes were placed inside a flask with 50 mL of a phosphate buffered solution isotonized with NaCl (pH 7.4). The flask was kept on a magnetic stirrer and stirring was maintained at 100 rpm at 33°C. 1 mL of release sample was withdrawn at pre-set times (5 min, 15 min, 30 min, once every hour over a period of 8 and 24 hours). The release of 5MCANAT was analyzed by HPLC following the above mentioned method.

#### 8. *In vivo* tear osmolarity measurements

This study was restricted to formulations F3 (5-MCA-NAT-loaded liposomes dispersed in 0.2% sodium hyaluronate) and F4 (5-MCA-NAT-loaded liposomes dispersed in 0.5% carboxymethylcellulose), that provided the highest *in* *vivo* hypotensive effect among all the tested formulations. An isotonic saline solution was used as control. Each formulation (control included) was tested in both eyes of three nonsedated male New Zealand white rabbits (n = 6 eyes).

Tear osmolarity was measured with the TearLab Osmolarity System (TearLab Corporation, San Diego, CA, USA). The device, stored in a temperature and humidity controlled room, was re-calibrated using electronic check cards at the beginning of each test session following the manufacturer’s guidelines. Tear samples of 50 nL were collected from the lower lateral meniscus of the eyes. To avoid fluctuation of tear osmolarity, all the measurements were made between 9 and 11 AM. Both eyes of rabbits were used for the evaluations. Measurements were taken before the administration of 25 µL of the formulation and 5, 15, 30 and 60 min after instillation.

#### 9. *In vivo* short-term tolerance study

This study was restricted to formulations F3 (5-MCA-NAT-loaded liposomes dispersed in 0.2% sodium hyaluronate) and F4 (5-MCA-NAT-loaded liposomes dispersed in 0.5% carboxymethylcellulose), that provided the highest *in* *vivo* hypotensive effect among all the tested formulations.

The experiment was carried out on both eyes of six male New Zealand rabbits. A single administration of 25 µL of the formulation were administered in the right eye of the animal. The contralateral eye was used as control and being instilled with the same volume of an isotonic solution of NaCl (pH 7.4).

Clinical symptoms and signs were evaluated in accordance with a protocol described previously [27]. Ocular surface evaluation was made before the instillation of the formulation or control, and then 1, 8 and 24 h after their administration. The short-term tolerance was evaluated by macroscopic examination of the eye surface of the animal and graded from 0–2, indicating the absence or presence of the following clinical signs: loss of corneal transparency, conjunctival signs (hyperemia, edema), eyelid swelling, and intense blinking (what would show a lack of tolerance).

#### 10. Statistical analysis

Intraocular hypotensive reduction was expressed as means ± standard error of the means (SEM). Other parameters as means ± standard deviation (SD) were also evaluated. Statistical differences between two mean values were evaluated by two-tailed Students t-test. If necessary, an analysis of variance (ANOVA) was employed. Results were taken as significantly different at p-values less than 0.05.

## Results

### 1. 5-MCA-NAT dose determination

The HPLC method to quantify 5-MCA-NAT was validated with respect to linearity, accuracy, and reliability in the range of concentrations between 5 and 50 µg/mL. In all cases, the method allowed sufficient separation of the drug from the vehicle. The retention time of 5-MCA-NAT was 10.7±0.5 minutes. There were non-significant differences (p = 0.57) between the theoretical concentration of 5-MCA-NAT (100 µM, ∼27.53 µg/mL) and the experimental values obtained (28.5±1.07 µg/mL). The percentage of 5-MCA-NAT loaded in the liposomes was 7.9±3.5%, relative to the entire amount of drug in the liposomal formulation. Taking into consideration that the volume of the instilled formulation in rabbit eyes was 25 µL, the dose of 5-MCA-NAT administered was 0.71±0.04 µg.

### 2. pH, osmolarity, and viscosity

The pH and osmolarity were measured for all formulations, and the viscosity was measured for liposomal formulations F2–F5 (Table 2). Formulation F1, without liposomes, had an acidic pH of 5.6. The incorporation of lipid vesicles in formulation F2 produced an increase of pH to 6.9, a nearly neutral value. The incorporation of polymer in formulations F3, F4, and F5 produced a minor decrease in pH, with values of 6.4, 6.5, and 6.5 respectively. The osmolarity of formulations F1, F2, F3, and F4 were within the range of isotonicity (Table 2). Formulation F5, with an osmolarity value of 237±3.1 mOsm, was hypotonic. The lowest viscosity, 1.2 mPa·s, was obtained in F2 (Table 2), the liposomal formulation without polymers. The addition of sodium hyaluronate 0.2% (F3) and carboxymethylcellulose 0.5% (F4) increased the viscosity of the formulations to 2.0 mPa·s and 7.3 mPa·s, respectively. The liposomal formulation with poloxamer (F5) had the highest viscosity, 28.3 mPa·s.

**Table 2:** pH, osmolarity, and viscosity data of the 5-MCA-NAT formulations.

Formulation	Polymer	pH	Osmolarity (mOsm)	Viscosity (mPa·s)
F1	-	5.6±0.02	295.8±0.5	-
F2	-	6.9±0.03	303.8±0.2	1.2±0.1
F3	Sodium hyaluronate	6.4±0.02	307.1±0.3	2.0±0.04*
F4	Carboxymethylcellulose	6.5±0.01	294.2±0.5	7.3±0.1*
F5	Poloxamer	6.5±0.01	237.2±3.1*	28.3±1.4*

Data are expressed as means ± SD (n = 3).

*Significant differences with formulation F2 (p-value<0.05).

### 3. Mean particle size and zeta potential of liposomal formulations

**Figure 1:**
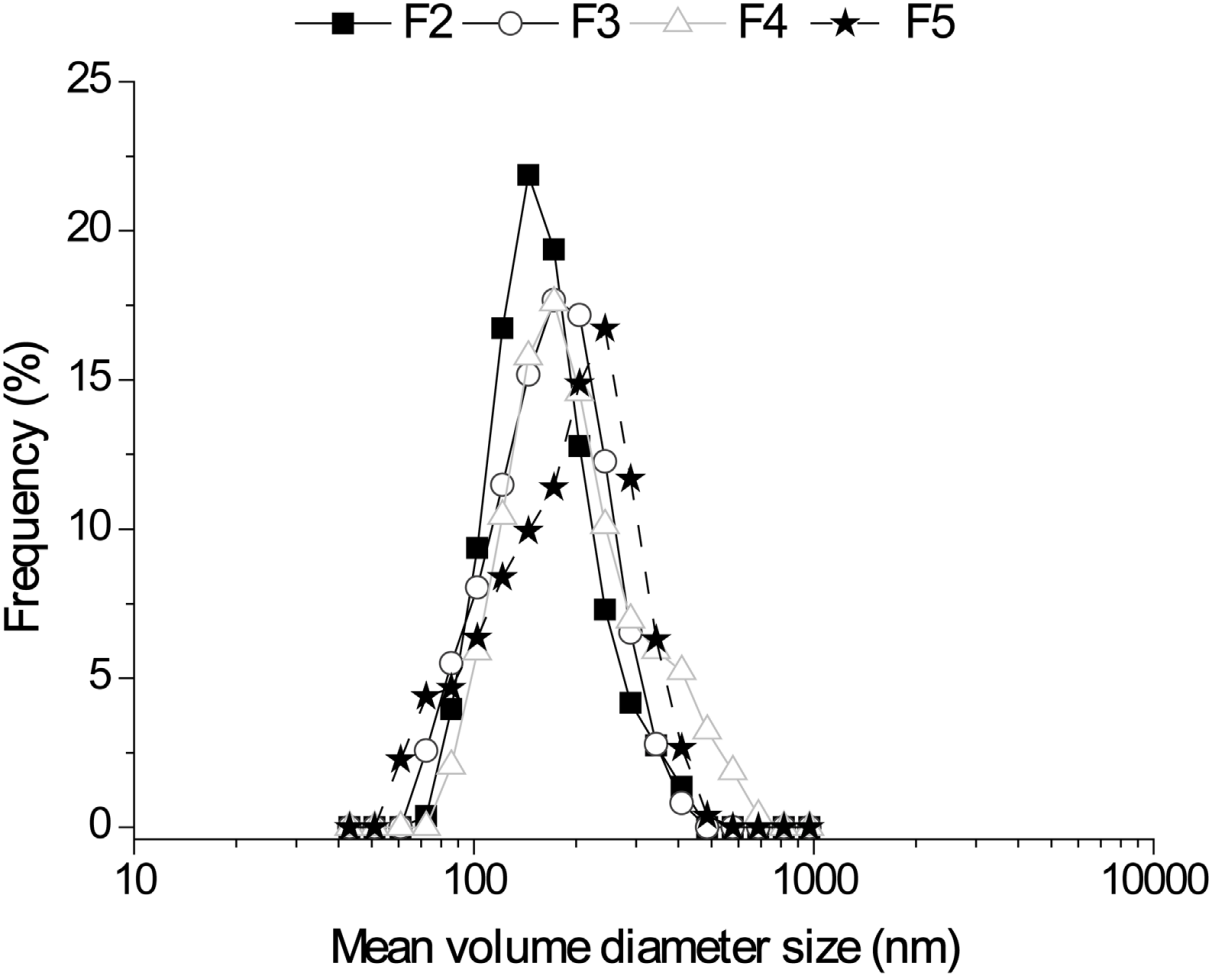
Size distribution of 5-MCA-NAT-loaded liposomes dispersed in NaCl (F2), 0.2% sodium hyaluronate (F3), 0.5% carboxymethylcellulose (F4) and 12/8 w/w poloxamer (F5).

All of the liposomal formulations showed unimodal size distributions (Figure 1), and the average particle sizes were less than 200 nm in all cases (Table 3). The average zeta potential was neutral (range between −10 to 10 mV) in all cases (Table 3).

**Table 3:** Mean diameter and zeta potential of the 5-MCA-NAT liposomal formulations.

Formulation	Polymer	Size (nm)	Zeta potential (mV)
F1	-	-	-
F2	-	155.1±4.0	10.2±2.7
F3	Sodium hyaluronate	162.8±7.7	8.7±2.7
F4	Carboxymethylcellulose	188.5±16.9*	7.2±4.5
F5	Poloxamer	181.8±12.8*	6.4±0.9

Data are expressed as means ± SD (n = 3).

*Significant differences with formulation F2 (p-value<0.05).

### 4. Effect of 5-MCA-NAT formulations on IOP in rabbits

**Figure 2:**
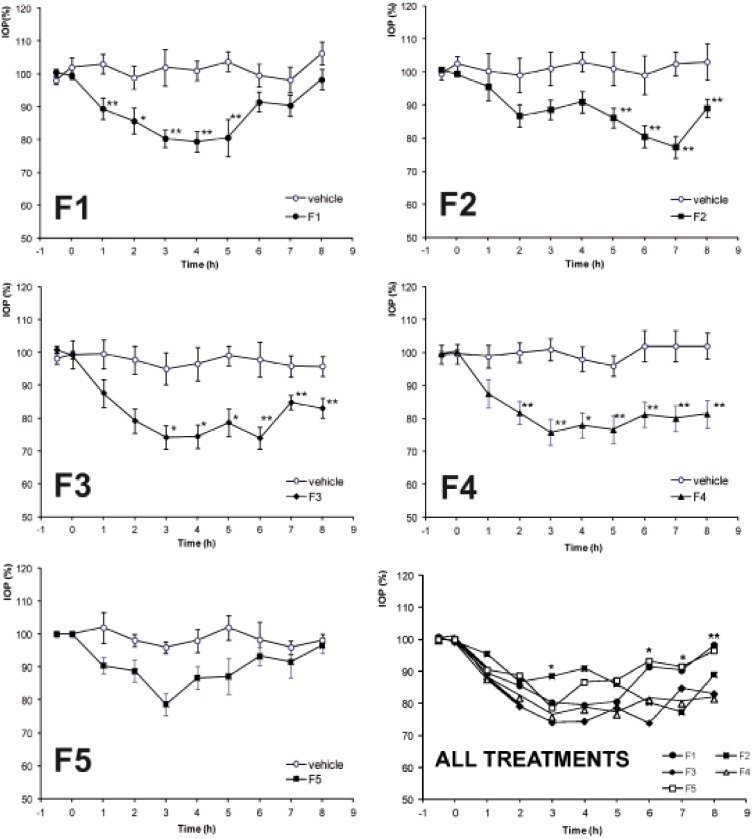
Ocular hypotensive effect of 5-MCA-NAT formulations. F1, solution of 5-MCA-NAT. F2, 5-MCA-NAT-loaded liposomes dispersed in NaCl; F3, 5-MCA-NAT-loaded liposomes dispersed in 0.2% sodium hyaluronate; F4, 5-MCA-NAT-loaded liposomes dispersed in 0.5% carboxymethylcellulose; F5, 5-MCA-NAT-loaded liposomes dispersed in 12/8 w/w poloxamer. Vehicles without 5-MCA-NAT were used as control in each case. Data are expressed as the mean ± SEM (n = 20).

All formulations reduced IOP in normotensive rabbits though the maximal effects were different for each formulation (Figure 2). The maximum percentage of IOP reduction, the area under the ΔIOP (%) time curve from 0 to 8 hours (estimated by the trapezoidal rule) and the duration of the hypotensive effect (h), were calculated for all the formulations (Table 4). In addition, several other IOP parameters were considered: mean of the minimum and maximum IOP, and mean difference between maximum and minimum IOP (Table 5). The IOP values measured were within the range of 8 to 16 mm Hg (Table 5). Formulation F3, composed of 5-MCA-NAT-loaded liposomes with 0.2% SH, produced the maximum hypotensive effect (Figure 2, Tables 4 and 5). This formulation provided an IOP reduction of 39.13±2.21%. The second highest value, 36.72±2.77%, was achieved by F4, composed of 5-MCA-NAT-loaded liposomes with 0.5% CMC. There were no significant differences between F3 and F4 with regard to the lowering of IOP (p = 0.55). The rest of the formulations provided significantly lower values (p<0.01 in all cases). For formulations F2, F3 and F4, the hypotensive effect was maintained for longer than 8 hours (p<0.05 for each formulation compared with the corresponding vehicle) (Figure 2 and Table 4). For F5, composed of 5-MCA-NAT-loaded liposomes dispersed in PX, the hypotensive effect lasted only 5 hours. Differences in the IOP can be observed among the formulations at 4 time points: 3, 6, 7 and 8 hours (p<0.05) although these differences are not systematically generated by the same formulation. Regarding the area under the ΔIOP (%) time curve from 0 to 8 hours, AUC_0_^8 h^, the highest value was shown with F3 (156.15±14.71), while the lowest with F5 (85.20±16.83).

**Table 4:** Maximum percent IOP reduction over 8 hours.

Formulation	Polymer	Maximum IOPreduction (%)	AUC_0_^8 h^ (%·h)	Duration (h)
F1	-	29.27±2.42	118.02±14.62	7
F2	-	29.44±2.40	112.07±15.93	+8
F3	Sodium hyaluronate	39.13±2.21*	156.15±14.71*	+8
F4	Carboxymethylcellulose	36.72±2.77*	131.84±16.81*	+8
F5	Poloxamer	29.12±2.11	85.20±16.83	5

IOP, intraocular pressure; Maximum IOP reduction (%), percentage of reduction ± SEM; AUC_0_^8 h^, ΔIOP (%) versus time (h) from 0 to 8 hours. Data are expressed as means ± SEM (n = 20 eyes).

*Significant differences with formulation F2 (p-value<0.05).

**Table 5:** IOP parameters over 8 hours.

Formulation	IOP_max_	IOP_min_	ΔIOP
F1	15.5±1.8	10.1±1.8	5.4±1.7
F2	14.7±1.8	9.7±1.4	5.0±1.6
F3	16.2±1.9	9.3±1.5	6.9±1.8
F4	14.7±3.0	8.6±1.5	6.1±2.5
F5	13.2±0.5	8.9±1.1	4.3±1.3

IOP, intraocular pressure in mmHg, IOP_max_, mean maximum IOP; IOP_min_, mean minimum IOP; ΔIOP, mean difference between IOP_max_ and IOP_min_. Data are expressed as means ± SEM (n = 20 eyes).

### 5. *In vitro* release studies

**Figure 3:**
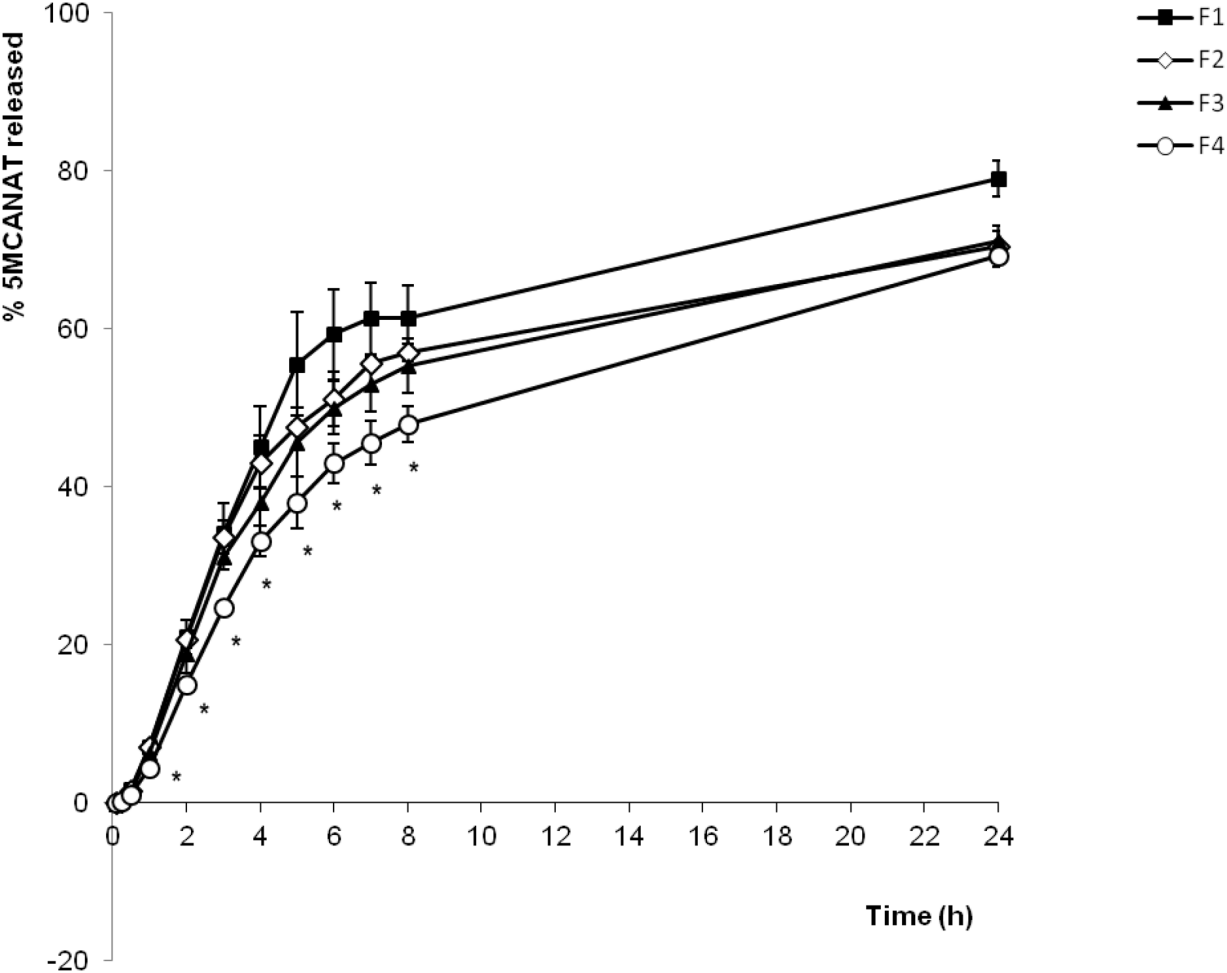
*In vitro* release curve of 5-MCA-NAT formulations in phosphate buffered solution at 33°C. F1: solution of 5-MCA-NAT; F2: 5-MCA-NAT-loaded liposomes dispersed in NaCl; F3: 5-MCA-NAT-loaded liposomes dispersed in 0.2% sodium hyaluronate; F4: 5-MCA-NAT-loaded liposomes dispersed in 0.5% carboxymethylcellulose. * Significant differences between formulations F2–F4 and formulation F1 (reference, p-value<0.05).

The *in* *vitro* release behavior of the solution of 5-MCA-NAT (F1), 5-MCA-NAT-loaded liposomes dispersed in NaCl (F2), 5-MCA-NAT-loaded liposomes dispersed in 0.2% SH (F3) and 5-MCA-NAT-loaded liposomes dispersed in 0.5% CMC (F4), is summarized in the cumulative percentage release shown in figure 3.

The release rate of the drug was higher for the solution of 5-MCA-NAT (F1), while the rate of release from 5-MCA-NAT-loaded liposomes dispersed in SH (F3) or CMC (F4) was lower than that of conventional liposomes (F2). Statistically significant differences between the solution of 5-MCA-NAT (F1) and liposomal formulations (F2–F4) were found after 1 hour of the beginning of the study (p<0.05 at all the time points studied). Comparing liposomal formulations, non-significant differences were found between conventional liposomes (F2) and liposomes dispersed in SH (p>0.25 in all cases). In the case of F4, 5-MCA-NAT released from liposomes resulted significantly lower to the ones that were found for the solution of 5-MCA-NAT (F1) or for conventional liposomes (F2) after 1 hour of the beginning of the study and up to 8 hours (p<0.003 in all cases). From 6 to 8 hours, a significant different release rate can be observed between F3 and F4, being F4 (5-MCA-NAT-liposomes dispersed with CMC) the formulation that showed a slower release profile (p<0.05).

### 6. *In vivo* tear osmolarity

Following the guidelines for the use of experimental animals, the number of rabbits was diminished by selecting the formulations that showed the best *in* *vivo* hypotensive effect: 5-MCA-NAT-loaded liposomes dispersed in 0.2% SH (F3) and 5-MCA-NAT-loaded liposomes dispersed in 0.5% CMC (F4). Results are collected in table 6.

**Table 6:** Tear osmolarity in the 3 Study Groups.

t (min)	Control Group	F3 Group	F4 Group
0	325.2±3.8	324.3±2.8	323.7±1.7
5	311.3±3.0	314.9±3.3*	316.7±1.0**
30	326.7±3.6	314.0±2.9*	313.2±3.4*
60	324.6±4.1	315.4±3.6*	311.8±1.9**

Control Group: animals were administered 25 µL of saline solution in both eyes; F3 Group animals were instilled 25 µL of 5-MCA-NAT-loaded liposomes dispersed in 0.2% SH (F3) in both eyes; F4 Group: animals received 25 µL of 5-MCA-NAT-loaded liposomes dispersed in 0.5% CMC (F4). Data are expressed as means ± SD (n = 6).

*Significant differences with Control Group (p-value<0.05).

**Significant differences with Control Group (p-value<0.01).

Instillation of a single drop of saline solution (Control Group) decreased the tear osmolarity for 5 min. However, 30 and 60 min later, osmolarity values were not significantly different to basal (p = 0.12). When animals were treated with liposomes combined with bioadhesive polymers (F3 and F4) the tear osmolarity diminished significantly at any time in comparison to basal (p = 0.04 and p = 0.0026, respectively).

### *7. In vivo* tolerance study

Before testing, all animals had a normal ocular surface and corneal transparency. None had any conjunctival disorders including hyperemia or edema, eyelid swelling, or intense blinking (grade 0). Animals showed no discomfort or irritation during the test or within 24 h after the administration of formulations F3 and F4, which contained 5-MCA-NAT-loaded liposomes dispersed in 0.2% SH and 0.5% CMC, respectively. The cornea remained transparent (no vessels) throughout the assay (grade 0) and the coloration of the conjunctiva remained normal (grade 0). No animal presented signs of mucus secretion.

## Discussion

In general, topical ophthalmic anti-glaucoma therapies combine the use of one or more hypotensive drugs with several instillations per day in chronic treatments. In these patients, the eye surface is continuously exposed to drugs and preservatives that produce, in most cases, ocular surface alterations and generally lead to a therapeutic failure [28]. Eye drops for chronic treatments should include components that are well tolerated for the ocular surface and, at the same time, enable enhanced drug bioavailability [29], [30]. In general, toxicity of formulations can be reduced by removing preservatives from the composition. However, in some cases the toxic effect of the drug still remains [31], [32], [33]. The inclusion of bioadhesive polymers have demonstrated to increase the tolerance of formulations while extending the effect of hypotensive drugs such as timolol maleate [34]. Nevertheless, the benefits produced by these polymers in terms of efficacy are limited, so it becomes necessary to use other technological approaches, such as combination of the drugs with other colloidal systems. Liposomes have gained considerable attention for ocular drug delivery. They have been primarily investigated as a modality to enhance corneal drug absorption through the ability to come into intimate contact with the corneal and conjunctival surfaces, thereby increasing the ocular drug penetration [17].

In this study, we designed novel liposomal formulations loaded with 5-MCA-NAT, a melatonin derivative that reduces IOP [12], [15]. The liposomes that we employed (150–200 nm, 6–10 mV zeta potential) simulate the lipid composition of the pre-ocular tear film and have demonstrated to be effective in the treatment of the dry eye syndrome [18]. The anti-glaucoma formulations of the current study were designed to (a) control the delivery of 5-MCA-NAT, thus prolonging the hypotensive effect, and (b) replenish the tear film, which may improve an ocular surface damaged by chronic exposure to topical treatments. To achieve the objectives, we prepared liposomes with the biocompatible components PC, Cht, and vitE – all of which are present in the natural tear film – that were loaded with 100 µM 5-MCA-NAT. Once prepared, the liposomes were dispersed in an isotonic aqueous solution alone or with the bioadhesive biopolymers SH or CMC, or a thermosensitive polymer, PX, to determine whether or not the use of these combinations would enhance the hypotensive effect of 5-MCA-NAT. The bioadhesive polymers and concentrations were selected among the available existing commercial artificial tear products [35]. A conventional solution of 100 µM 5-MCA-NAT without liposomes was used as the reference.

All the formulations developed satisfied the given requirements of pH and osmolarity for ophthalmic solutions [36]. Regarding pH values, the presence of lipid vesicles in formulation F2 produced a slight increase of pH to a nearly neutral value, probably due to the presence of PC in the formulation. The incorporation of polymers in formulations F3, F4, and F5 produced minor decreases in pH, presumably due to the acidic nature of SH, CMC and PX. For the formulations F2–F5, the pH values were nearly neutral, thus helping to maintain the optical properties of the eye surface, epithelial cell functions, and cellular homeostasis. With respect to the osmolarity, all of the formulations were within an acceptable range for ophthalmic administration [23].

To obtain a homogeneous film after instillation, the viscosity of a formulation should be similar to that of natural tears, 1–9 mPa·s [37], [38]. Previous studies showed that the most significant relative improvement in ocular bioavailability occurred for vehicles in the viscosity range from 1 to 15 mPa·s [39], [40]. In our study, the viscosities of formulations with bioadhesive polymers were within this range, and the most effective formulations for reducing IOP, F3 and F4, had viscosity values of 2.0 mPa·s and 7.3 mPa·s, respectively. The formulation F5 had the highest viscosity, 28.3 mPa·s, but it was no more effective in reducing IOP than were the non-liposomal F1 or the liposomal F2 without a bioadhesive polymer. This anomalous behavior of F5 (liposomes dispersed with Poloxamer) could be associated with its high viscosity and sol–gel phase transition phenomena [41].

The bioadhesive properties and rheological behavior of SH and CMC are widely described [42], [43]. Mucins present in the pre-ocular tear film can interact with the bioadhesive polymers and increase the residence time of the formulation on the eye surface and improve drug bioavailability. Formulations F2 (5-MCA-NAT-liposomes), F3 (5-MCA-NAT-liposomes dispersed with SH) and F4 (5-MCA-NAT-liposomes dispersed with CMC) present hypotensive effect up to 8 hours after a single administration unlike formulation F1 (5-MCA-NAT dissolved in 0.788% NaCl and 0.275% PG). This effect can be attributed to possible interactions between components of the formulations (liposomes and bioadhesive polymers) with the ocular surface (mucins and/or tear film). *In vitro* release studies also corroborate these results. Formulation F4 (5-MCA-NAT-liposomes dispersed with CMC) shows significant differences in the release rate from 6 h to 8 h with the rest of formulations and a tendency to extend the hypotensive effect in animals. Taking into account that hypertensive animals show higher IOP decrease after the use of hypertensive drugs (in comparison to normotensive animals), an induced hypertensive animal models might be useful to appreciate differences in the extension of the hypotensive effect [44], [45].

In addition, these polymers hydrate and protect the ocular surface by forming a film that covers the eye. So, the liposomes dispersed with bioadhesive polymers contribute to the modulation of drug delivery by adequately mixing with the natural tears that cover the ocular surface. The liposomes also reduce water evaporation from tears and help improve symptoms such as dryness, redness, and visual impairment associated with dry eyes. The mucoadhesive behavior of these hybrid formulations was exhibited when rabbit eyes’ tear osmolarity decreased to reach osmolarity values similar to the formulations F3 and F4 that were maintained for at least 60 minutes after a single instillation. These results must be confirmed in humans due to the physiological differences between both species that can affect the residence time of the formulations on the eye surface (i.e. lower blink rate than humans). In any case, further studies are necessary to demonstrate the efficacy of these formulations, in terms of ocular surface damage, that require experimental animal models.

The goal of the study was to enhance the effect of the hypotensive agent 5-MCA-NAT by using an unpreserved drug delivery system that could increase its bioavailability while being soft with the ocular surface. The novel hybrid formulations simulate tear film composition and include biocompatible components that might relieve symptoms of secondary diseases affecting the ocular surface derived from the chronic use of anti-glaucoma topical medications. With these improvements, patient adherence to topical glaucoma therapy could be enhanced.

## Conclusions

A number of applications of liposomes in ophthalmic drug delivery have been extensively studied [46], [47], [48], [49], [50]. The improvement in the precorneal retention, transcorneal permeation, and therapeutic efficacy has been explored in detail, providing information about the interaction between liposomes and ocular tissues. In this work we prepared novel liposomal formulations dispersed in aqueous solutions of bioadhesive or amphiphilic block copolymers for the delivery of the ocular hypotensive agent 5-MCA-NAT. *In vivo* efficacy studies in rabbits showed that the hypotensive effect of the drug was remarkably increased with the combination of liposomes and bioadhesive polymers and also tear osmolarity decreased significantly for 60 min. This improvement could be attributed to the influence of liposomes as drug carriers, the increased residence time of the formulation on the eye surface derived from the mucoadhesive properties of the polymers and the ability of these hybrid formulations to simulate and replenish the tear film. Addition of the thermosensitive polymer poloxamer to the drug-loaded liposomes was no more effective than the non-liposomal 5-MCA-NAT. In this work, the application of liposomes combined with bioadhesive polymers for the improvement of the precorneal retention of hypotensive drugs has shown potential for further investigation. In conclusion, hybrid nanosystems composed by liposomes combined with bioadhesive polymers might serve as potential ocular drug carriers that prolong drug retention and improve biocompatibility of the formulations on the ocular surface.
